# [^68^Ga]Pentixafor-PET/CT for imaging of chemokine receptor 4 expression in small cell lung cancer - initial experience

**DOI:** 10.18632/oncotarget.7063

**Published:** 2016-01-28

**Authors:** Constantin Lapa, Katharina Lückerath, Martina Rudelius, Jan-Stefan Schmid, Alexander Schoene, Andreas Schirbel, Samuel Samnick, Theo Pelzer, Andreas K. Buck, Saskia Kropf, Hans-Jürgen Wester, Ken Herrmann

**Affiliations:** ^1^ Department of Nuclear Medicine, University Hospital Würzburg, Würzburg, Germany; ^2^ Institute for Pathology, University of Würzburg, Würzburg, Germany; ^3^ Department of Internal Medicine, Caritas Hospital Bad Mergentheim, Bad Mergentheim, Germany; ^4^ Department of Internal Medicine, University Hospital Würzburg, Würzburg, Germany; ^5^ Scintomics GmbH, Fürstenfeldbruck, Germany; ^6^ Pharmaceutical Radiochemistry, Technische Universität München, Munich, Germany; ^7^ Department of Molecular and Medical Pharmacology, David Geffen School of Medicine at UCLA, Los Angeles, CA, USA

**Keywords:** small cell lung cancer, SCLC, molecular imaging, CXCR4, PET

## Abstract

Chemokine receptor CXCR4 is a key factor for tumor growth and metastasis in several types of human cancer. This study investigated the feasibility of CXCR4-directed imaging of small cell lung cancer (SCLC) with positron emission tomography/computed tomography (PET/CT) using the radiolabelled chemokine ligand [^68^Ga]Pentixafor.

10 patients with primarily diagnosed (n=3) or pre-treated (n=7) SCLC (n=9) or large cell neuroendocrine carcinoma of the lung (LCNEC, n=1) underwent [^68^Ga]Pentixafor-PET/CT. 2-[^18^F]fluoro-2-deoxy-D-glucose ([^18^F]FDG, n=6) and/or somatostatin receptor (SSTR)-directed PET/CT with [^68^Ga]DOTATOC (n=5) and immunohistochemistry (n=10) served as standards of reference.

CXCR4-PET was positive in 8/10 patients and revealed more lesions with significantly higher tumor-to-background ratios than SSTR-PET. Two patients who were positive on [^18^F]FDG-PET were missed by CXCR4-PET, in the remainder [^68^Ga]Pentixafor detected an equal (n=2) or higher (n=2) number of lesions. CXCR4 expression of tumor lesions could be confirmed by immunohistochemistry.

Non-invasive imaging of CXCR4 expression in SCLC is feasible. [^68^Ga]Pentixafor as a novel PET tracer might serve as readout for confirmation of CXCR4 expression as prerequisite for potential CXCR4-directed treatment including receptor-radio(drug)peptide therapy.

## INTRODUCTION

Small cell lung cancer (SCLC) is a neuroendocrine tumor that represents 15% of all lung cancers [1]. It occurs predominantly in smokers as almost all patients have a history of tobacco use.

SCLC is distinguished clinically from most other types of non-small cell lung cancer by its rapid doubling time, high growth fraction, and the early development of metastases. Only one-third of patients are diagnosed with localized disease, and treatment strategies have focused on systemic therapy [2]. Although SCLC is highly responsive to both chemotherapy and radiotherapy, it commonly relapses within months despite treatment [3, 4]. Response rates to second-line treatments have been reported to range between 10-20% [5]. Thus, new treatment options including personalized medicine targeting specific molecular markers are urgently needed.

Chemokine receptor CXCR4 has been described to play a pivotal role in tumor growth and progression, tumor invasiveness and metastasis [6]. Overexpression of the receptor has been reported in more than 30 different types of cancer, including lymphoma, breast, pancreatic, ovarian and lung cancer [7-10]. In SCLC, almost ubiquitous CXCR4 overexpression has been shown to correlate with negative outcome [11].

Recently, a radiolabelled CXCR4-ligand ([^68^Ga]Pentixafor) for PET imaging has been developed [12, 13]. Dosimetry and proof-of-concept for visualization of CXCR4-expression has recently been demonstrated in patients with hematologic malignancies [14, 15], glioblastoma [16] and myocardial infarction [17, 18].

This is the first report of non-invasive detection of CXCR4-expression in SCLC patients. CXCR4 may serve as a promising new target for both diagnostic and therapeutic interventions, especially in selecting potential candidates for endoradiotherapy.

## RESULTS

### Clinical findings

8/10 patients presented with extended disease, 2 subjects with limited disease. Metastatic sites included lymph nodes (9/10), adrenals (4/10), liver (4/10), pleura (3/10), bone (2/10), and brain (1/10). Intrapulmonary metastases were detected in (3/10) subjects, 1 patient suffered from leptomeningeal tumor spread. All pretreated patients had undergone chemotherapy with cisplatin and etoposide. At the time point of imaging, only 2/10 patients were currently on treatment. Patient #2 had started combined radio-chemotherapy 14 days prior to [^68^Ga]-Pentixafor-PET. In patient #5, chemotherapy had been initiated 9 days earlier.

### Imaging results

[^68^Ga]Pentixafor-PET was visually positive in 8/10 patients. Interestingly, the only patient with LCNEC (patient #6) did not demonstrate any relevant [^68^Ga]Pentixafor uptake. The negative SCLC patient (patient #2) was on combined radio-chemotherapy which had been initiated 14 days prior to imaging. All other patients exhibited intense tracer accumulation in both primary tumor as well as lymph node and organ metastases.

### Patient-basis analysis

**Figure 1:**
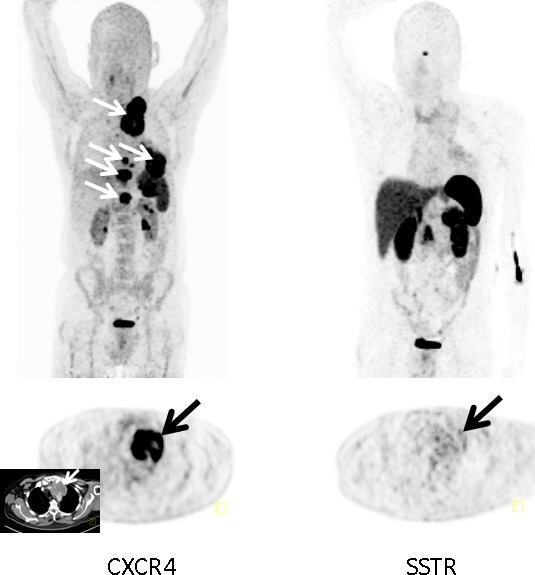
Example of a CXCR4-positive, SSTR-negative SCLC patient Display of maximum intensity projections (upper row) and transaxial images (lower row) of both CXCR4- and SSTR-directed PET/CT (interval between both scans, 6 days) in a patient with recurrent SCLC (patient #3). Whereas [^68^Ga]Pentixafor-PET demonstrates intense tracer retention in various tumor manifestations including mediastinal lymph nodes, bone and pleural lesions, SSTR-directed PET proves negative (arrows; insert: corresponding contrast-enhanced transaxial CT).

In comparison to [^68^Ga]DOTATOC (*n* = 5), 2/5 subjects were [^68^Ga]Pentixafor-positive/SSTR-negative (Figure 1), 2/5 patients were positive and 1/5 negative in both scans.

In comparison to [^18^F]FDG (*n* = 6), both [^68^Ga]Pentixafor and [^18^F]FDG were positive in 4/6 patients. The remaining two patients presented with [^18^F]FDG-positive/CXCR4-negative lesions.

### Lesion-basis analysis

On a lesion basis, in the 5 patients in whom [^68^Ga]DOTATOC-PET was performed, [^68^Ga]Pentixafor detected a total of 55 lesions (lymph nodes, *n* = 33; liver, *n* = 9; pleura, *n* = 6; brain, *n* = 4; adrenals, *n* = 2; lungs, *n* = 1) whereas SSTR-PET depicted only 20 foci (lymph nodes, *n* = 14; adrenals, *n* = 2; brain, *n* = 2; pleura, *n* = 1; lungs, *n* = 1). The 35 lesions exclusively visualized by [^68^Ga]Pentixafor were lymphonodal (*n* = 19), hepatic (*n* = 9), pleural (*n* = 5) and CNS (*n* = 2) manifestations of origin; 16 of those were present in the two CXCR4-positive/SSTR-negative subjects.

**Figure 2:**
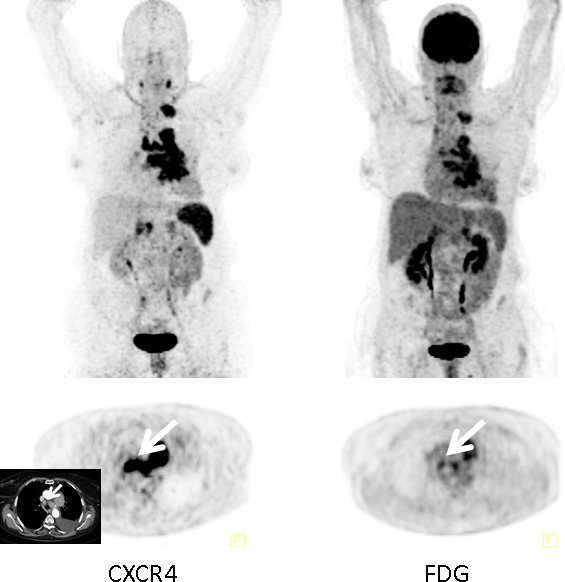
Example of additional value of [^**68**^Ga]Pentixafor-PET in an SCLC patient Display of maximum intensity projections (upper row) and transaxial images (lower row) of both CXCR4- and [^18^F]FDG-PET/CT (interval between both scans, 6 days) in a patient with recurrent SCLC (patient #4). [^68^Ga]Pentixafor-PET demonstrates more intense tracer retention in various tumor manifestations including mediastinal lymph nodes (arrows; insert: corresponding contrast-enhanced transaxial CT).

In comparison to [^18^F]FDG (44 lesions), CXCR4-directed PET visualized 33 tumor manifestations. 23 foci were missed in the two [^18^F]FDG-positive/[^68^Ga]Pentixafor-negative patients. In the remaining 4 subjects, CXCR4-PET detected 26 lymph node (*vs*. [^18^F]FDG: *n* = 15), 3 adrenal (*vs*. [^18^F]FDG: *n* = 3), 3 lung (*vs*. [^18^F]FDG: *n* = 3), and 1 liver (*vs*. [^18^F]FDG: *n* = 0) lesions/metastases (Figure 2).

On semi-quantitative analysis, the median SUV_mean_ of the primary tumors was 6.9 (range, 2.6-11.3) and the median SUV_max_ was 8.8 (range, 4.8-15.5). The corresponding SUV_mean_ for [^68^Ga]DOTATOC and [^18^F]FDG were 5.6 (range, 2.7-8.5) and 7.1 (range, 3.1-24.6), respectively; those for SUV_max_ 9.1 (range, 4.7-13.5; [^68^Ga]DOTATOC) and 9.9 (range, 4.7-38.1; [^18^F]FDG). The respective value for the hottest metastatic lesion in [^68^Ga]Pentixafor-PET were 7.5 (range, 3.0-14.1) for SUV_mean_ and 10.0 (range, 6.5-19.4) for SUV_max_, respectively. This compared to 9.4 (range, 8.9-9.9; [^68^Ga]DOTATOC) and 7.0 (range, 3.4-15.5; [^18^F]FDG) for SUV_mean_ and for SUV_max_ to 17.6 (range, 16.7-18.5; [^68^Ga]DOTATOC) and 11.2 (range, 4.9-19.3; [^18^F]FDG). Tumor-to-background ratios were significantly higher for [^68^Ga]Pentixafor than for [^68^Ga]DOTATOC regarding the primary tumor as well as the hottest metastatic lesion with a median P/B_mean_ of 5.4 (range, 1.3-7.5) *vs*. 0.7 (range, 0.3-1.0) and a median P/B_max_ of 3.7 (range, 1.8-5.1) *vs*. 0.7 (range, 0.4-1.0). Median M/B_mean_ ratios were 5.5 (range, 2.4-12.9) *vs*. 1.2 (range, 1.1-1.2) and median M/B_max_ ratios were 3.2 (range, 2.4-8.3) *vs*. 1.5 (range, 1.3-1.6), respectively. [^68^Ga]Pentixafor ratios were comparable to [^18^F]FDG (median P/B_mean_, 3.0; range, 1.4-8.6; median P/B_max_, 3.2; range, 1.4-8.7; median M/B_mean_, 4.1; range, 1.5-6.7; median M/B_max_, 3.5; range, 1.5-8.6). The individual SUV and tumor-to-background values for CXCR4 are given in Supplementary Table 1.

### Immunohistochemistry

**Figure 3:**
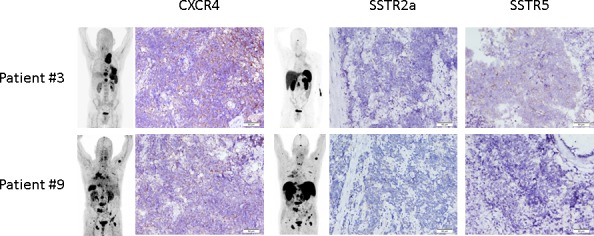
Immunohistochemical expression of CXCR4 and somatostatin receptors 2a and 5 in SCLC Display of two examples of immunohistochemical expression of CXCR4 and SSTR2a and 5, respectively. Patient #3 had his biopsy taken from a lymph node metastasis demonstrating a weak staining for CXCR4 in 90% of the tumor cells (IRS 4). SSTR2a was negative, SSTR5 could also be demonstrated to be weakly expressed in 90% (IRS 4). Patient #9 also presented with extensive disease. Biopsy of the primary tumor revealed mild CXCR4 (intensity 1+ in 70% of the cells, IRS 3) and mild SSTR2a (intensity 1+ in 90%; IRS 4) expression. SSTR5 was negative in the sample. The inserts depict maximum intensity projections are the respective whole-body [^68^Ga]Pentixafor- and SSTR-directed PET/CT scans, respectively.

In all patients imaging results could be compared to immunohistological staining for SSTR2a/5 and CXCR4 derived from biopsies of the primary tumor (*n* = 6) or metastases (*n* = 4) (Figure 3). Regarding the histological evaluation of CXCR4 expression, 1/10 samples was rated “mild” (IRS 3), 6/10 “moderate” (4 IRS 4, 2 IRS 8) and 2/10 “strongly” (1 IRS 10, 1 IRS 12) positive. The remaining sample (patient #5) was scored negative. In comparison, 5/10 samples did not show any SSTR2a/5 expression (all IRS 0), and 5/10 were moderately (all IRS 4) positive (Table 1).

**Table 1:** Patients' characteristics

patient	sex	age (y)	disease	stage	previous Tx	site of Bx	Bx to PET	PentixaforPET/CT	DOTATOCPET/CT	FDGPET/CT	CXCR4IRS	SSTR2IRS	SSTR5IRS
**1**	m	69	SCLC	ED	RCTx	primary	2 months	+	+	n/a	8	0	0
**2**	f	38	SCLC	ED	none	metastasis	1 week	−	n/a	+	10	0	4
**3**	m	63	SCLC	ED	RCTx	metastasis	3 weeks	+	-	n/a	4	0	4
**4**	f	62	SCLC	ED	CTx	primary	4 weeks	+	n/a	+	4	0	0
**5**	f	49	SCLC	LD	none	primary	2 weeks	+	n/a	+	0	0	0
**6**	f	74	LCNEC	ED	RCTx	metastasis	5 months	−	−	+	12	0	0
**7**	m	63	SCLC	ED	RCTx	metastasis	2 weeks	+	−	n/a	4	4	4
**8**	m	56	SCLC	ED	RCTx	primary	8 months	+	n/a	+	8	0	0
**9**	m	79	SCLC	ED	CTx	primary	2 months	+	+	n/a	3	4	0
**10**	f	67	SCLC	LD	none	primary	2 weeks	+	n/a	+	4	4	0

All patients with relapsed disease had undergone 1^st^- and/or 2^nd^-line treatment (patients #1, #3, #9). 1^st^-line treatment included 2-6 cycles of platinum/etoposide-containing chemotherapy (patient #1: 2 cycles, patients #3 and #9: 4 cycles, patients #4, #6, #7 and #8: 6 cycles) (+radiotherapy), 2^nd^-line therapy had topotecan as the mainstay (all three patients [#1, #3 and #9] received 6 cycles).

Bx = biopsy; CTx = chemotherapy; IRS = immunoreactive score; LCNEC = large cell neuroendocrine carcinoma of the lung; LN = lymph node; N/A = not assessed; PD= primary diagnosis; RCTx = radio-chemotherapy; SCLC= small cell lung cancer; Tx = treatment.

Immunohistological CXCR4 score did not correspond to the intensity of [^68^Ga]Pentixafor uptake (SUV_mean_, SUV_max_, TBR_mean_, TBR_max_). Interestingly, two patients (#2, #6) had no detectable CXCR4-expression by PET/CT, whereas receptor expression was maximal on all tumor cells (IRS 10 and 12, respectively). Patient #6 had the biopsy sample taken from a cervical lymph node 5 months prior to PET imaging and received 4 cycles of carboplatin and etoposide in the interim; patient #2 had started radio-chemotherapy 14 days prior to PET-imaging. The other patient receiving chemotherapy (patient #5, therapy initiated 9 days prior to PET imaging) presented with negative histology for CXCR4 but positive [^68^Ga]Pentixafor-PET.

## DISCUSSION

This is the first report of *in vivo* imaging of CXCR4 expression in humans with both newly diagnosed as well as pre-treated, recurrent SCLC. A recent report evaluating biopsy samples of bronchopulmonary neuroendocrine tumors demonstrated a high intensity of CXCR4 receptor expression in SCLC. Additionally, chemokine receptor expression was a predictor of poor overall survival [11]. In concordance, moderate to high receptor density on the cell surface was visualized by PET/CT in our cohort in the vast majority of cases.

Of note, in comparison to [^18^F]FDG-PET/CT as reference, almost all tumor lesions proved to be CXCR4-positive with high tumor-to-background ratios, thus rendering CXCR4 a promising target for endoradiotherapy. Given the high relapse rate of SCLC after 1^st^ line chemotherapy as well as the modest response rates to subsequent treatments, a new approach to the patient with relapsed/refractory SCLC is urgently needed. In addition to conventional chemotherapeutic agents including anthracycline-based regimes, topotecan or amrubicin [19], peptide receptor radionuclide therapy using radiolabelled somatostatin receptor ligands has been performed with rather modest success rates [20, 21]. Important prerequisites to receptor-targeted therapies are a robust expression of the target receptor as well as a specific receptor binding. In our patient cohort, CXCR4-PET/CT clearly outperformed SSTR-PET on a patient as well as a lesion basis, underlining the potential superiority of CXCR4 as a therapeutic target compared to SSTR.

Recently, a derivative of the diagnostic compound allowing labelling with various α- and β-emitters called Pentixather has been developed. Proof-of-concept for endoradiotherapy could be demonstrated in patients with advanced multiple myeloma with partial and complete metabolic responses [22]. Thus, further assessment of this promising tool in a theranostic approach is warranted.

This pilot study has several limitations. First, only a limited number of patients could be included in the study. Second, biopsies were not always obtained on a short-term period compared to the time point of PET imaging and re-biopsies could not always be acquired. [^68^Ga]Pentixafor uptake did not seem to correlate with histological receptor expression, maybe due to receptor kinetics and internalization. Of note, receptor surface expression of CXCR4 seems to be a dynamic process which is influenced by a number of factors including therapeutic agents. The patient demonstrating the highest CXCR4 expression in her sample analyzed (patient #6) presented with a negative [^68^Ga]Pentixafor PET after concurrent 1^st^ line chemotherapy. In addition, another patient (patient #2) with recently diagnosed SCLC also did not exhibit high receptor expression on PET after initiation of combined radio-chemotherapy two weeks earlier. Therefore, one might speculate that surface expression of CXCR4 is downregulated as a response to treatment in a time and dose dependent manner. In line with this observation is the fact that another subject (patient #5) showed intermediate SUV and no immunohistochemical CXCR4-positivity after a nine-day-duration of chemotherapy. While high expression of CXCR4 in SCLC has recently been demonstrated [11], future studies to further investigate therapy-induced down- and -preferably- up-regulation of CXCR4 are warranted. Potentially a sequential combination with chemotherapeutic agents with might lead to improved efficacy of CXCR4-directed endoradiotherapy.

In conclusion, our data demonstrate the feasibility of [^68^Ga]Pentixafor for PET imaging of CXCR4 chemokine receptor expression in SCLC patients. This novel PET tracer might serve as an innovative imaging agent for *in vivo* biomarker identification that could result in patient selection for CXCR4-directed treatment, and, eventually, for receptor-radio(drug)peptide therapy.

## MATERIALS AND METHODS

### Subjects and study design

From January to September 2015, 10 patients (5 males and 5 females, mean age, 62±12 years: range, 38-79 years) with newly diagnosed (*n* = 3) or pretreated (*n* = 7) SCLC (*n* = 9) or large cell neuroendocrine carcinoma (LCNEC) (*n* = 1) underwent imaging with [^68^Ga]Pentixafor-PET. Routine staging or restaging examinations included [^18^F]FDG (*n* = 6) or somatostatin receptor (SSTR) -directed PET with [^68^Ga]DOTATOC (*n* = 5; the latter for identification of candidates for SSTR-directed therapy). Patients' characteristics are given in more detail in Table 1.

[^68^Ga]Pentixafor was administered on a compassionate use basis in compliance with §37 of the Declaration of Helsinki and The German Medicinal Products Act, AMG §13 2b and in accordance with the responsible regulatory body (Regierung von Oberfranken). The study adhered to the standards established in the declaration of Helsinki. All patients gave written informed consent prior to imaging.

### Imaging

[^68^Ga]Pentixafor was prepared as previously described [23]. In short, all syntheses were performed in a fully automated, GMP-compliant procedure using a GRP^®^ module (SCINTOMICS GmbH, Germany) equipped with a disposable single-use cassette kit (ABX, Germany). The eluate (^68^Ga^3+^ in 0.6 M HCl) of a ^68^Ge/^68^Ga-generator (iThemba Labs, Faure, South Africa) was transferred to a cation exchange cartridge, eluted with 5 N NaCl, added to a solution of 40 μg Pentixafor (Scintomics, Fürstenfeldbruck, Germany) in HEPES-buffer and heated for 6 minutes at 125°C. The product was immobilized on a SepPak C18-cartridge, washed with water und eluted with ethanol/water 50/50. The eluate was passed through a sterile filter (0.22 μm) into a sterile vial und diluted with phosphate buffer solution to a total volume of 15 mL. Radiochemical purity was determined by gradient high performance liquid chromatography and thin-layer chromatography. Additionally, the product was also tested for ethanol content, pH, radionuclide purity, sterility, and endotoxins.

[^18^F]FDG was synthesized in house with a 16 MeV Cyclotron (GE PETtrace 6; GE Healthcare, Milwaukee, USA). [^68^Ga]DOTATOC was prepared using a modification of the method described by Breeman et al. [24] using a SCINTOMICS radiotracer synthesis module (Scintomics, Fürstenfeldbruck, Germany).

All PET scans ([^18^F]FDG, [^68^Ga]DOTATOC and [^68^Ga]Pentixafor) were performed on a dedicated PET/CT scanner (Siemens Biograph mCT 64; Siemens Medical Solutions, Germany) within two weeks. Before acquisition of FDG-PET scans, patients fasted for at least 6 h prior to injection of [^18^F]FDG. Blood glucose levels were < 160 mg/dl. Prior to [^68^Ga]DOTATOC and [^68^Ga]Pentixafor scans, no fasting was necessary. Imaging was perfomed 60 minutes after injection of 124 to 149 MBq (median, 142 MBq) of [^68^Ga]Pentixafor, 229 to 304 MBq (median, 297 MBq) of [^18^F]FDG and 120 to 164 MBq (median, 157 MBq) of [^68^Ga]DOTATOC, respectively. Corresponding CT low-dose scans for attenuation correction were acquired using a low-dose protocol (20 mAs, 120 keV, a 512 × 512 matrix, 5 mm slice thickness, increment of 30 mm/s, rotation time of 0.5 s, and pitch index of 0.8) including the base of the skull to the proximal thighs. Consecutively, PET emission data were acquired in three-dimensional mode with a 200 × 200 matrix with 2-3 min emission time per bed position. After decay and scatter correction, PET data were reconstructed iteratively with attenuation correction using a dedicated software (Siemens Esoft, Siemens, Erlangen, Germany).

### Image analysis

All PET scans were first visually rated by a board-certified nuclear medicine physician in a binary visual fashion as positive for disease or negative for disease. Semi-quantitative analysis was performed for the primary as well as the hottest metastatic lesion. The axial PET image slice displaying the maximum tumor uptake was selected by drawing a 3D-volume of interest (VOI) around the whole tumor area. Tumor regions of interest (ROIs) were defined in 2 ways. First, a standardized 10-mm circular region was placed over the area with the peak activity. This first ROI was used to derive maximum (SUV_max_) and mean standardized uptake values (SUV_mean_). A reference region was defined by drawing a ROI (diameter of 50 mm) involving normal liver parenchyma to derive tumor-to-background ratios. Both primary-to-background (P/B) as well as metastasis-to-background (M/B) ratios for SUV_max_ and SUV_mean_ were calculated. The radiotracer concentration in the ROIs was normalized to the injected dose per kilogram of patient's body weight to derive the SUVs.

### Immunohistochemistry

Immunohistochemistry was carried out on 10% formalin fixed paraffin embedded tissue sections (3μm) according to established protocols and scored as described previously (13). CXCR4-immunohistochemistry was performed using an anti-CXCR4 rabbit polyclonal antibody (ab2074; Abcam, Cambridge, UK) followed by detection with the DAKO en vision system according to the manufacturer's protocol.

For assessment of SSTR expression, polyclonal antibodies against SSTR2A (1:500, RBK 046-05, Zytomed, Berlin, Germany) and SSTR5 (1:500, RBK 051-05, Zytomed, Berlin, Germany) were used. Samples from normal pancreatic tissue were used as positive control (islet cells). Dewaxed samples were pretreated with citrate buffer pH 6.0 for 10 minutes (for SSTR2a staining) or with the antigen retrieval agent TIRS-EDTA pH 9.0, respectively, for 10 minutes in a high pressure cooker (for SSTR5 staining). All immunostained sections were counterstained for 3 minutes with hematoxylin. The analysis of the stained sections was done semi-quantitatively by light-microscopy according to the immunoreactive score (IRS) by Remmele and Stegner [25]. The percentage of CXCR4-/SSTR-positive cells was scored as follows: 0 (no positive cells), 1 (< 10% positive cells), 2 (10-50% positive cells), 3 (> 50-80% positive cells), 4 (> 80% positive cells). Additionally, the intensity of staining was graded: 0 (no color reaction), 1 (mild reaction), 2 (moderate reaction), 3 (intense reaction). Multiplication of both scores for a given sample yields the IRS classification: 0-1 (negative), 2-3 (mild), 4-8 (moderate), 9-12 strongly positive.

### Statistical analysis

Most of the data are descriptive. Statistical analyses were performed using IBM SPSS (version 22.0; SPSS, Inc. Chicago, IL). Quantitative values were expressed as mean ± standard deviation or median and range as appropriate. Comparisons of related metric measurements were performed using Mann-Whitney-U test. A *p*-value < 0.05 was considered to indicate statistical significance.

## SUPPLEMENTARY TABLE



## References

[1] Govindan R, Page N, Morgensztern D, Read W, Tierney R, Vlahiotis A, Spitznagel EL, Piccirillo J (2006). Changing epidemiology of small-cell lung cancer in the United States over the last 30 years: analysis of the surveillance, epidemiologic, and end results database. Journal of clinical oncology.

[2] Cuffe S, Moua T, Summerfield R, Roberts H, Jett J, Shepherd FA (2011). Characteristics and outcomes of small cell lung cancer patients diagnosed during two lung cancer computed tomographic screening programs in heavy smokers. Journal of thoracic oncology.

[3] Wolfson AH, Bae K, Komaki R, Meyers C, Movsas B, Le Pechoux C, Werner-Wasik M, Videtic GM, Garces YI, Choy H (2011). Primary analysis of a phase II randomized trial Radiation Therapy Oncology Group (RTOG) 0212: impact of different total doses and schedules of prophylactic cranial irradiation on chronic neurotoxicity and quality of life for patients with limited-disease small-cell lung cancer. International journal of radiation oncology, biology, physics.

[4] Foster NR, Qi Y, Shi Q, Krook JE, Kugler JW, Jett JR, Molina JR, Schild SE, Adjei AA, Mandrekar SJ (2011). Tumor response and progression-free survival as potential surrogate endpoints for overall survival in extensive stage small-cell lung cancer: findings on the basis of North Central Cancer Treatment Group trials. Cancer.

[5] Fruh M, De Ruysscher D, Popat S, Crino L, Peters S, Felip E, Group EGW (2013). Small-cell lung cancer (SCLC): ESMO Clinical Practice Guidelines for diagnosis, treatment and follow-up. Annals of oncology.

[6] Domanska UM, Kruizinga RC, Nagengast WB, Timmer-Bosscha H, Huls G, de Vries EG, Walenkamp AM (2013). A review on CXCR4/CXCL12 axis in oncology: no place to hide. European journal of cancer.

[7] Burger JA, Kipps TJ (2006). CXCR4: a key receptor in the crosstalk between tumor cells and their microenvironment. Blood.

[8] Burger JA, Peled A (2009). CXCR4 antagonists: targeting the microenvironment in leukemia and other cancers. Leukemia.

[9] Muller A, Homey B, Soto H, Ge N, Catron D, Buchanan ME, McClanahan T, Murphy E, Yuan W, Wagner SN, Barrera JL, Mohar A, Verastegui E, Zlotnik A (2001). Involvement of chemokine receptors in breast cancer metastasis. Nature.

[10] Zhang B, Wu T, Wang Z, Zhang Y, Wang J, Yang B, Zhao Y, Rao Z, Gao J (2015). p38MAPK activation mediates tumor necrosis factor-alpha-induced apoptosis in glioma cells. Molecular medicine reports.

[11] Kaemmerer D, Reimann C, Specht E, Wirtz RM, Sayeg M, Baum RP, Schulz S, Lupp A (2015). Differential expression and prognostic value of the chemokine receptor CXCR4 in bronchopulmonary neuroendocrine neoplasms. Oncotarget.

[12] Demmer O, Gourni E, Schumacher U, Kessler H, Wester HJ (2011). PET imaging of CXCR4 receptors in cancer by a new optimized ligand. ChemMedChem.

[13] Wester HJ, Keller U, Schottelius M, Beer A, Philipp-Abbrederis K, Hoffmann F, Simecek J, Gerngross C, Lassmann M, Herrmann K, Pellegata N, Rudelius M, Kessler H, Schwaiger M (2015). Disclosing the CXCR4 expression in lymphoproliferative diseases by targeted molecular imaging. Theranostics.

[14] Philipp-Abbrederis K, Herrmann K, Knop S, Schottelius M, Eiber M, Luckerath K, Pietschmann E, Habringer S, Gerngross C, Franke K, Rudelius M, Schirbel A, Lapa C, Schwamborn K, Steidle S, Hartmann E (2015). *In vivo* molecular imaging of chemokine receptor CXCR4 expression in patients with advanced multiple myeloma. EMBO molecular medicine.

[15] Herrmann K, Lapa C, Wester HJ, Schottelius M, Schiepers C, Eberlein U, Bluemel C, Keller U, Knop S, Kropf S, Schirbel A, Buck AK, Lassmann M (2015). Biodistribution and radiation dosimetry for the chemokine receptor CXCR4-targeting probe 68Ga-pentixafor. Journal of nuclear medicine.

[16] Lapa C, Lückerath K, Kleinlein I, Monoranu C, Linsenmann T, Kessler A, Rudelius M, Kropf S, Buck A, Ernestus R, Wester H, Löhr M, Herrmann K (2016). 68Ga-Pentixafor-PET/CT for imaging of chemokine receptor 4 expression in glioblastoma. Theranostics.

[17] Thackeray JT, Derlin T, Haghikia A, Napp LC, Wang Y, Ross TL, Schafer A, Tillmanns J, Wester HJ, Wollert KC, Bauersachs J, Bengel FM (2015). Molecular Imaging of the Chemokine Receptor CXCR4 After Acute Myocardial Infarction. JACC Cardiovascular imaging.

[18] Lapa C, Reiter T, Werner RA, Ertl G, Wester HJ, Buck AK, Bauer WR, Herrmann K (2015). [(68)Ga]Pentixafor-PET/CT for Imaging of Chemokine Receptor 4 Expression After Myocardial Infarction. JACC Cardiovascular imaging.

[19] von Pawel J, Jotte R, Spigel DR, O'Brien ME, Socinski MA, Mezger J, Steins M, Bosquee L, Bubis J, Nackaerts K, Trigo JM, Clingan P, Schutte W, Lorigan P, Reck M, Domine M (2014). Randomized phase III trial of amrubicin *versus* topotecan as second-line treatment for patients with small-cell lung cancer. Journal of clinical oncology.

[20] Sollini M, Farioli D, Froio A, Chella A, Asti M, Boni R, Grassi E, Roncali M, Versari A, Erba PA (2013). Brief report on the use of radiolabeled somatostatin analogs for the diagnosis and treatment of metastatic small-cell lung cancer patients. Journal of thoracic oncology.

[21] Pless M, Waldherr C, Maecke H, Buitrago C, Herrmann R, Mueller-Brand J (2004). Targeted radiotherapy for small cell lung cancer using 90Yttrium-DOTATOC, an Yttrium-labelled somatostatin analogue: a pilot trial. Lung cancer.

[22] Herrmann K, Schottelius M, Lapa C, Osl T, Poschenrieder A, Haenscheid H, Lueckerath K, Schreder M, Bluemel C, Knott M, Keller U, Schirbel A, Samnick S, Lassmann M, Kropf S, Buck A (2015). First-in-man experience of CXCR4-directed endoradiotherapy with 177Lu- and 90Y-labelled pentixather in advanced stage multiple myeloma with extensive intra- and extramedullary disease. Journal of nuclear medicine.

[23] Martin R, Juttler S, Muller M, Wester HJ (2014). Cationic eluate pretreatment for automated synthesis of [(6)(8)Ga]CPCR4.2. Nuclear medicine and biology.

[24] Breeman WA, de Blois E, Sze Chan H, Konijnenberg M, Kwekkeboom DJ, Krenning EP (2011). (68)Ga-labeled DOTA-peptides and (68)Ga-labeled radiopharmaceuticals for positron emission tomography: current status of research, clinical applications, and future perspectives. Seminars in nuclear medicine.

[25] Kaemmerer D, Peter L, Lupp A, Schulz S, Sanger J, Baum RP, Prasad V, Hommann M (2012). Comparing of IRS and Her2 as immunohistochemical scoring schemes in gastroenteropancreatic neuroendocrine tumors. International journal of clinical and experimental pathology.

