# Supplementary material for: [68Ga]Pentixafor-PET/CT for imaging of chemokine receptor 4 expression in small cell lung cancer - initial experience
Source: Oncotarget. 2016 Jan 28;7(8):9288–95. doi: 10.18632/oncotarget.7063 (PMC4891040; doi:10.18632/oncotarget.7063)
Supplement: Supplementary file 1 [file oncotarget-07-9288-s001.pdf]

[^{68}Ga]Pentixafor-PET/CT for imaging of chemokine receptor 4 expression in small cell lung cancer - initial experience

Supplementary Material

Supplementary Table 1: SUV_{mean}, SUV_{max}, primary-to-background (P/B), hottest metastasis-to-background (M/B) ratios as well as immunoreactive scores (IRS) for [^{68}Ga]Pentixafor-PET

	Primary		Primary/Bkg		Metastasis		Metastasis/Bkg		CXCR4 -IRS
	**SUV_{mean}**	**SUV_{max}**	**P/B_{mean}**	**P/B_{max}**	**SUV_{mean}**	**SUV_{max}**	**M/B_{mean}**	**M/B_{max}**	
**1**	7.0	8.8	5.5	3.8	7.4	10.0	5.8	4.4	8
**2**	neg	neg	neg	neg	neg	neg	neg	neg	10
**3**	6.9	8.6	6.3	3.7	14.1	19.4	12.9	8.3	4
**4**	11.3	15.5	7.5	5.1	11.1	18.1	7.4	6.0	4
**5**	6.0	9.3	4.7	3.7	4.2	6.5	3.2	2.6	0
**6**	neg	neg	neg	neg	neg	neg	neg	neg	12
**7**	n/a	n/a	n/a	n/a	3.0	6.9	2.4	2.4	4
**8**	2.6	4.8	1.3	1.8	5.4	8.6	2.8	3.1	8
**9**	5.2	8.7	3.6	2.0	8.9	13.3	6.2	3.1	3
**10**	8.0	10.8	5.4	3.6	7.7	9.8	5.2	3.3	4
